# Recurrence of Dysentery

**Published:** 1854-04

**Authors:** 


Recurrence of Dysentery.—Dr. A. F. Jetter, of Palmyra, Mo., has not found, with Dr. Flint, that second attacks of Dysentery are things of rare occurrence. He thus writes in the *St. Louis Medical and Surgical Journal:*

"In 1846, I was the subject of an attack of dysentery, and judging from the amount of pain attendant, thought it a severe case. In 1847, however, I had an attack more violent and protracted than the one of the former year. Since that season I have practiced every year, where it prevailed epidemically—during which time I have had four additional attacks, two of which were of a character so grave as to render recovery for a time doubtful. So you will perceive, I have six several times in my own person, and in different seasons, suffered severe and unequivocal attacks of dysentery. Although I have kept no record of the number of cases, yet more than thirty instances of 'recurrence' of dysentery among my neighbors and acquaintances, now occur to my memory, in some of whom the disease has occurred three or four several times. I have recently (Nov. 10, 1853,) attended four members of a family severally afflicted with dysentery, all of whom I treated for the same disease, two years ago, and another member of the same family living in an adjoining county, has had the disease every summer for the last five. Inasmuch as the attack I had in 1847 was worse than the one of the preceding year, I was for a time inclined to the belief, that one occurrence of dysentery might render each succeeding attack worse; but farther acquaintance with the complaint has not confirmed the opinion; but that one attack does not destroy our susceptibility to the 'recurrence' of dysentery, let the foregoing facts declare."

